# A Treatise on Pathological Anatomy

**Published:** 1844-12

**Authors:** 


Art. IX. *A Treatise on Pathological Anatomy.* By Carl Rokitansky, M.D. Professor Extraordinary of Pathological Anatomy of the University of Vienna. Part I. Containing the abnormal conditions of the Organs of Respiration. Translated from the German, with additions on Diagnosis, from Shoenlein, Skoda, and others. By Dr. John C. Peters. New York: Wm. Radde. 1845. 8 vo. p.p. 164.

The first number of this work, which we have received from the Translator, is accompanied by testimonials as to its great excellence from some of the most competent judges in this country, and some of the most authoritative journals abroad. Among the former are Professors Parker and Mott; and of the latter, the British and Foreign Medical Review holds, concerning it, the following language: "Dr. Rokitansky's book is no more than it professes to be: it is morbid anatomy in its densest and most compact form, scarely ever alleviated by histories, cases, or hypotheses." The opportunities of the author for wide and accurate observation appear to have been of the best kind, and he gives evidence of having improved them with true German industry. This work is said to contain the result of his experience gained in the examination of over twelve thousand dead bodies. To the pathological Anatomy of Rokitansky, the Translator has added copious notes on *Diagnosis*, from Schoenlein and Skoda, who belong to the same school of laborious observers, and of whom we are told, that the former is the most original and accurate auscultator and percussor, and the latter the greatest master of general diagnosis, in Germany. Rokitansky is a remarkable man, and as his name is probably new to nearly every reader of this Journal, we subjoin a brief sketch of his professional training and character, which we find in the preface of the Translator. Dr. Peters, who was a pupil of the learned German, says—

"It is well to add, that Rokitansky was first educated as a hospital physician; next, he was trained up in the dead-house in the celebrated Sifft, and finally was elected to the professorship of pathological anatomy; that all medico-legal, or so-called coroner's cases, are submitted to him for examination, so that life and death hang upon his verdict almost every day, and he is forced to acquire a most extended knowledge of practical medicine, which is the more necessary to him as he is daily called upon to verify or condemn the diagnoses of numerous clinical professors. Such is the training to which his naturally researchful and truthful impulses have been subjected through a long series of years. Right honestly and modestly does he perform his delicate duties; ever inclining to the side of mercy, he is firm enough to add the weight of his testimony to that which has been accumulated against a trembling or hardened criminal, and never is found weak enough or mean enough to gloss over the mistakes or flatter the vanity of hospital physicians."

The Translator seems to have bestowed uncommon labor upon his task. He says:

"We are scarcely ashamed to own that the present is the fourth version which we have made; twice we essayed the task unaided, and although we had read the work repeatedly and carefully, and were famillar with Rokitansky's style, from hearing his lectures and enjoying familiar private intercourse with him, we could not succeed in making even a passable translation. We had thrown up the task in despair, although our admiration and friendship for Rokitansky had drawn us to it as to a "labor of love," and we had received not only his permission but his request to undertake it, when Dr. Wotherspoon volunteered his assistance and almost forced us to the third trial. Even the result of our joint labors did not meet a very low ideal of what the work ought to be, and hence we not only revised and compared it with the original text, but re-wrote the whole. We pledged ourselves for the faithfulness of the translation, and although Dr. Wotherspoon took considerable pains to render the style more English, it will still be found foreign and rude in many respects."

The style is eminently condensed, and that in a scientific work is a virtue broad enough to cover a multitude of venial offences. The appearance of the book is good: the paper is white, firm and thick, and the letter, one upon which the eye reposes with satisfaction. We hope the Translator will be encouraged to go on with the work to its completion. That will depend upon the sale with which the part now before the public meets. This part is perfect in itself, and will constitute a valuable monograph on the abnormal condition of the organs of respiration, should the remaining parts never appear.

---

Art. X.—*The Cyclopædia of Practical Medicine.* Edited by John Forbes, M.D., F.R.S., Alex. Tweedie, M.D., F.R.S., and John Conolly, M.D. Revised, with additions, by Robley Dunglison, M.D. Parts XIII., XIV., and XV. Philadelphia: Lea & Blanchard. 1844.

With the first of these numbers begins the third volume of the Cyclopædia, which will be completed with the twenty-fourth, now in a few months. In conversation with practising physicians, we have been gratified to find, that this work comes fully up to the high expectations formed of it from the complimentary notices in the journals, and that as a work of reference it is regarded as superior to anything hitherto published on practical medicine.

